# Retrospective analysis of 1,203 cases of referral to a quaternary vascular surgery outpatient clinic within the Unified Health System, São Paulo, Brazil

**DOI:** 10.31744/einstein_journal/2024AO0676

**Published:** 2024-05-09

**Authors:** Felipe Soares Oliveira Portela, Carlos Augusto Rossetti, Thulio Fernandes de Souza, Arthur Souza Magnani, Marcelo Fiorelli Alexandrino da Silva, Maria Fernanda Cassino Portugal, Marcelo Passos Teivelis, Nelson Wolosker, Cynthia de Almeida Mendes

**Affiliations:** 1 Hospital Israelita Albert Einstein São Paulo SP Brazil Hospital Israelita Albert Einstein, São Paulo, SP, Brazil.; 2 Faculdade Israelita de Ciências da Saúde Albert Einstein Hospital Israelita Albert Einstein São Paulo SP Brazil Faculdade Israelita de Ciências da Saúde Albert Einstein, Hospital Israelita Albert Einstein, São Paulo, SP, Brazil.

**Keywords:** Patient-referral systems, Tertiary care centers, Vascular surgical procedures, Public health, Telemedicine

## Abstract

**Objective:**

Consequently, in this study, we aimed to evaluate 1,203 cases of referral to a quaternary vascular surgical service, in São Paulo, Brazil, over a 6-year period, to assess the appropriate need for referral; in addition to the prevalence of surgical indications.

**Methods:**

In this retrospective analysis, we reviewed the institutional records of participants referred from Basic Healthcare Units to a vascular surgical service inside the Brazilian Unified Health System, between May 2015 and December 2020. Demographic and clinical data were collected. The participants were stratified, as per the reason for referral to the vascular surgical service, previous imaging studies, and surgical treatment indications. Referral appropriateness and complementary examinations were evaluated for each disease cohort. Finally, the prevalence of cases requiring surgical treatment was defined as the outcome measure.

**Results:**

Of the 1,203 referrals evaluated, venous disease was the main reason for referral (53%), followed by peripheral arterial disease (19.4%). A considerable proportion of participants had been referred without complementary imaging or after a long duration of undergoing an examination. Referrals were regarded as inappropriate in 517 (43%) cases. Of these, 32 cases (6.2%) had been referred to the vascular surgical service, as the incorrect specialty. The percentage of referred participants who ultimately underwent surgical treatment was 39.92%. Carotid (18%) and peripheral arterial diseases (18.4%) were correlated with a lower prevalence of surgical treatments.

**Conclusion:**

The rate of referral appropriateness to specialized vascular care from primary care settings was low. This may represent a subutilization of quaternary surgical services, with low rates of surgical treatment.

## In Brief

Through a retrospective analysis of 1,203 cases of referral from primary healthcare units to a specialized quaternary vascular surgical service, the findings of this study revealed a high proportion of inappropriate referrals, which may represent a substantial subutilization of this highly complex service.

## Highlights

Forty-three percent of referrals from basic healthcare units to a specialized vascular surgical service were regarded as inappropriate.

The main reasons for inappropriate referrals included study participants without an indication for surgery and those referred to the wrong specialty.

A high proportion of inappropriate referrals resulted in the subutilization of highly complex services.

Use of telemedicine will assist in reducing the rate of inappropriate referrals.

## Graphical abstract

**Figure:**
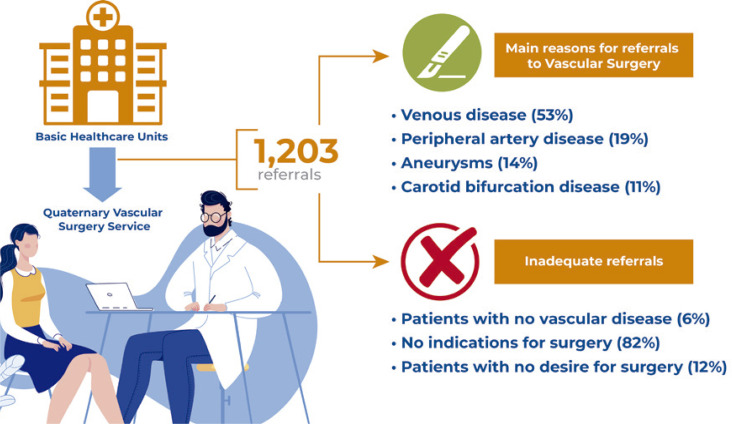

## INTRODUCTION

The Brazilian Unified Health System (SUS - *Sistema Único de Saúde*) is one of the world’s most extensive public health systems, encompassing primary, secondary, and tertiary care.^(1)^Since the early 1980s, several advancements have been made in the social sphere of Latin America. The creation of the Brazilian SUS is part of these developments and focuses on the expansion of universal healthcare and assistance.^(2)^

Currently, approximately 71.5% of the Brazilian population is exclusively covered by the public system.^(3)^ Within the SUS, a patient must first be evaluated by a general practitioner (GP) to be appropriately referred to a specialized, tertiary institution when needed. Determining the possible barriers that may hinder or delay patients’ access to appropriate care is a priority for improving public healthcare efficiency.^(4)^

One of the most practical aspects of the system’s operation is patient referral among the several healthcare levels, which is potentially the aspect with the most substantial impact on the perceptions of the patients concerning this system.

Regarding the proper referral within the SUS system, a patient must be admitted to one of the entry points, the Basic Health Units, Emergency Services, or Psychosocial Services, where most primary health care demands are met.

Regarding the more complex cases, referral is frequently required at the higher secondary, tertiary, or quaternary healthcare levels, at which more advanced examinations, procedures, and specialized attention are available.^(5)^

Despite being well-founded in theory, data permitting the evaluation of this referral protocol are scarce. A study conducted in a capital city of the southern region of Brazil has revealed that 41.7% of neurological referrals from the SUS Basic Healthcare Units could have been resolved at the primary care level, without a need for further specialized evaluation.^(6)^ Such patients, who have been unnecessarily referred, occupy hospital beds and overload the system.^(7)^

Few studies have evaluated the appropriateness of specialized referrals within healthcare systems. A 2011 systematic review has evaluated 214 studies, which included only 13 focusing on specialized referral appropriateness.^(8)^ In particular, fewer studies have focused on assessing referrals to surgical services. To the best of our knowledge, previous studies have not evaluated cases of referral to quaternary vascular surgical services.

## OBJECTIVE

Thus, in this study, we aimed to evaluate 1,203 cases of referral to a quaternary ambulatory vascular surgical service, in São Paulo, over a period of 6 years, to assess the appropriate need for referral regarding specialized evaluations; in addition to the prevalence of surgical indications.

## METHODS

### Ethical considerations

All data were collected from electronic records and were adequately de-identified. This study was approved by the research ethics committee of *Hospital Israelita Albert Einstein* (CAAE: 34994720.0.0000.0071; #4.321.559) and was conducted in accordance with the national legislation and principles of the Declaration of Helsinki for research on human participants. Informed consent was obtained from participants who were still undergoing outpatient follow-up during the data collection period.

### Study design

In this retrospective analysis, we reviewed the institutional records of all participants referred from SUS Basic Healthcare Units to an ambulatory vascular surgical service, between May 2015 and December 2020. This quaternary hospital and referral center for the surgical care of patients forms part of the SUS and is located in São Paulo, Brazil.

All ambulatory referrals from a Basic Healthcare Unit during the study period were included. Patients who had been previously evaluated by a vascular surgical service or were internally referred from within the hospital for ambulatory follow-up were excluded.

Data on the sex, age, height, weight, and body mass index were automatically collected as part of the consultation protocol. Additionally, data regarding comorbidities, medications, and medical and surgical histories were collected from the medical records.

Participants were stratified according to the reasons for referral to the vascular surgical service, availability of previous imaging studies and whether first-care attendants had requested them, surgical treatment indications, the inclination of the participants for surgical care, and referral appropriateness.

Thus, cases were divided into five primary vascular disease cohorts: 1) venous diseases, including chronic venous insufficiency, acute or subacute deep vein thrombosis, and post-thrombotic syndrome; 2) carotid arterial disease; 3) aneurysmal diseases, including aortoiliac aneurysms and peripheral or visceral artery aneurysms; 4) peripheral arterial disease (PAD); and 5) other vascular diseases, such as diabetic foot ulcers, arteriovenous fistulas, pseudoaneurysms, and miscellaneous diseases.

The availability of previous imaging studies was stratified as follows: 1) participants referred with no or inadequate examinations for suspected disease, 2) participants referred with adequate examinations performed >6 months before evaluation, and 3) participants referred with adequate and current examinations <6 months.

Adequacy of the examinations was categorized as follows: 1) a venous duplex scan of the legs, for the venous disease cohort; and 2) an arterial duplex scan, angiogram, angiotomography, or angioresonance for the carotid arterial disease, aneurysms, and PAD cohorts.

Each referral case was categorized as “appropriate” or “inappropriate,” as per the relevant need for specialized evaluation at a quaternary level. Participants appropriately referred to a quaternary vascular surgical service were those requiring specialized evaluation, after presenting with a vascular disease, with an indication for surgical intervention. Participants inappropriately referred to a quaternary vascular surgical service, were as follows: 1) referral to the incorrect specialty; 2) participants presenting with a vascular disease, however with no indication for surgical intervention that could be managed at a primary health care level; 3) participants presenting with a vascular disease, with indication for surgical intervention, who refused surgical treatment from the first encounter.

Moreover, the prevalence of each disease was assessed. The prevalence of the most common clinical manifestations and temporal distribution of the main reasons for referral were evaluated throughout the study period.

The referral appropriateness and complementary examinations were evaluated for each disease cohort. Furthermore, we defined the prevalence of cases in which surgical intervention was the outcome, for each disease cohort. An assessment of the surgical treatments by year has been performed.

### Statistical analysis

All data were compiled using Microsoft Office Excel 2016 (Redmond, WA, USA).

## RESULTS

Of the 1,203 participants, 1,150 and 21 had been referred, due to one of the main and less frequent vascular diseases, respectively. Furthermore, 32 participants had been incorrectly referred or had not presented with vascular conditions.

The sex distribution, prevalence of comorbidities, and average age at first evaluation of the participants are presented, by disease cohort, in table 1.

**Table 1:** Demographic characteristics of the participants by disease cohort

	Venous disease (n=635) n (%)	Carotid arterial disease (n=128) n (%)	Aneurysm (n=164) n (%)	PAD (n=223) n (%)	Total (n=1,150) n (%)
Sex					
Female	468 (73.7)	57 (44.5)	60 (36.5)	98 (43.9)	683 (59.4)
Male	167 (26.3)	71 (55.5)	104 (63.5)	125 (56.1)	467 (40.6)
Hypertension	257 (40.5)	111 (86.7)	131 (79.9)	167 (74.9)	666 (58.1)
DM	91 (14.4)	56 (43.8)	44 (26.8)	95 (42.6)	286 (24.7)
HF	8 (1.3)	16 (12.5)	16 (9.8)	23 (10.3)	63 (5.4)
Smoking (current or previous)	139 (21.8)	82 (64.0)	126 (76.8)	178 (79.8)	525 (45.5)
Dyslipidemia	49 (7.8)	26 (20.3)	28 (17.1)	45 (20.2)	148 (12.7)
Coronary disease	11 (1.7)	25 (19.5)	30 (18.3)	45 (20.2)	111 (9.7)
Myocardial revascularization	6 (0.9)	17 (13.3)	21 (12.8)	26 (11.7)	70 (6.0)
Non-dialysis chronic kidney disease	0	16 (12.5)	26 (15.9)	19 (8.6)	61 (5.3)
Hemodyalisis	0	0	1 (0.6)	0	1 (0.2)
Previous lower limb amputation	0	1 (0.8)	2 (1.2)	16 (7.2)	19 (1.7)
Stroke	8 (1.3)	66 (51.6)	19 (11.6)	27 (12.1)	120 (10.4)
TIA	1 (0.2)	18 (14.2)	0	0	19 (1.8)
Previous DVT	16 (2.5)	2 (1.6)	4 (2.4)	2 (0.9)	24 (2.1)
Average age at the first evaluation	55.7	69.1	69.6	65.4	61

PAD: peripheral arterial disease; DM: *diabetes mellitus*; HF: heart failure; TIA: transient ischemic attack; DVT: deep vein thrombosis.

Venous disease was the main cause of referral, accounting for 53% of all referred cases, followed by PAD (19.4%). Additionally, common cardiovascular risk factors were prevalent in our study population.

The availability and adequacy of complementary examinations, at the first evaluation are detailed in table 2. A considerable proportion of patients was referred without complementary imaging or after a long duration of undergoing an examination.

**Table 2:** Availability and adequacy of the complementary examinations at the first evaluation

	Venous disease (n=635) n (%)	Carotid arterial disease (n=128) n (%)	Aneurysms (n=164) n (%)	PAD (n=223) n (%)
No or inadequate examinations	72 (11.4)	3 (2.3)	4 (2.4)	84 (37.7)
Adequate examination >6 months	276 (43.5)	64 (50)	51 (31.1)	46 (20.6)
Adequate examination <6 months	286 (45.1)	61 (47.7)	109 (66.5)	93 (41.7)

PAD: peripheral arterial disease.

Of the participants with carotid arterial disease, 87.6% were asymptomatic, or had not presented with neurological manifestations in the previous 6 months. Of these participants, 10.2% presented with complete occlusion at the first evaluation, 52.3% presented with >70% stenosis, and 35.5% presented with ≤70% stenosis.

Referrals due to aneurysmal disease were primarily correlated with a dilatation of the infrarenal abdominal aorta (70.7%). The second most prevalent disease was popliteal aneurysms (8.5%), followed by iliac artery (7.9%), and thoracoabdominal aortic aneurysms (7.9%). Visceral artery and thoracic aortic aneurysms were the least prevalent, at 2.5% each.

Peripheral arterial disease cases of referral were mostly those involving claudication (64%); with only 36% of participants presenting with signs or symptoms of critical limb ischemia, such as rest pain, ulcers, or gangrene affecting the unilateral or bilateral lower limbs. Most (58.7%) participants presented with femoropopliteal stenotic lesions. Aortoiliac involvement was observed in 26.5% of the referred participants, while 14.5% had infrapopliteal lesions.

Referrals were regarded as inappropriate in 517 (43%) cases, including participants presenting with vascular diseases not requiring surgical intervention, participants not consenting to surgical treatment from the first encounter, or being incorrectly referred to the vascular surgical service.

The referral appropriateness, as per disease cohort is depicted in table 3. In absolute numbers, venous disease principally contributed to inappropriate referrals; while, proportionally, the most inappropriate referrals were attributed to PAD.

**Table 3:** Distribution of referral appropriateness by disease cohort

Reason for referral (n=1,203)	Appropriately referred cases n (%)	Inappropriately referred cases n (%)	Total n (%)
Venous disease (n=635)	460 (72.4)	175 (27.6)	635 (52.8)
Carotid arterial disease (n=128)	63 (49.2)	65 (50.8)	128 (10.6)
Aneurysms (n=164)	80 (48.8)	84 (51.2)	164 (13.7)
PAD (n=223)	69 (30.9)	154 (69.1)	223 (18.5)
Other vascular diseases (n=21)	14 (66.7)	7 (33.3)	21 (1.7)
Referrals to the wrong specialty (n=32)	0	32 (100)	32 (2.7)
Total	686 (56.5)	517 (43.5)	1,203 (100.0)

PAD: peripheral arterial disease.

Of the 517 inappropriate referrals, 32 (6.2%) had been referred to the incorrect specialty. In 426 (82.4%) cases, participants had been referred for surgical intervention; nonetheless, they had not presented with an indication thereof. The remaining 59 (11.4%) participants presented with vascular disease with surgical indications; however, they did not wish to undergo invasive procedures.

Regarding this last cohort, most (69.5%) participants were referred for chronic venous disease; while 16.9%, 8.5%, and 5.1% of participants were referred, due to aneurysms, carotid arterial disease, and PAD, respectively.

The percentage of participants who had been referred cases, and ultimately underwent surgical treatment was 39.92%. Table 4 presents the surgical treatments of the cases by year, with a tendency towards a decrease in surgical treatment for all referred cases.

**Table 4:** Proportions of surgical treatment for referred cases, by year

Year	Total of referred cases	Total of cases surgically treated	Proportion of surgical treatment (%)
2015	13	9	69.2
2016	327	177	53.5
2017	318	134	41.8
2018	190	63	33.2
2019	191	50	26.2
2020	83	15	18.1

Participants with carotid arterial disease and PAD had lower percentages of surgical treatment (18% and 18.4%, respectively). Conversely, participants with aneurysmal (49.4%) and venous diseases (47%) had higher percentages of surgical treatment.

## DISCUSSION

A simple, overall analysis of our data revealed that approximately half of the referrals did not require specialized quaternary care. This may have a huge impact on the health system at several levels, particularly regarding increased costs and delayed provision of care to patients who actually require specialized services.

Surgical indications for main vascular pathologies are well established. For instance, generally, patients with visible varicose veins should undergo surgical intervention, to avoid complications of chronic venous disease and improve symptoms. However, patients who are unwilling to undergo surgery may be conservatively managed with compressive therapy and adequate lifestyle changes, without the need for referral to specialized services.^(9)^

Regarding arterial aneurysms, the surgical indication is dependent on the dilatation diameter, growth rate, or symptom manifestation.^(10-13)^ Furthermore, cases not requiring surgical intervention could be conservatively followed up by a GP, until such time that an intervention is indicated.

Carotid arterial disease conventionally requires surgery when >70% stenosis is present, or when patients present with symptoms associated with >50% stenosis. Non-hemodynamically, clinically significant stenoses <50% and total occlusions counter-indicate invasive treatment^(14)^ and should be managed with optimized clinical therapy.

In cases of PAD, surgical treatment is indicated for critical limb ischemia, as evidenced by rest pain, ulcers, or gangrene. Additionally, surgery may be indicated in selected cases of limiting claudication, nonresponsive to 6 months of optimized clinical therapy.^(15)^ For stable claudication, the recommended treatment is optimized medical therapy,^(16)^ including lifestyle changes, risk factor control, aspirin, statins, and structured exercises.^(17,18)^

Determining referral appropriateness is a complex task.^(19)^ In this study, because the focus was on quaternary vascular surgical services, referrals were regarded as appropriate, when specialized surgical treatment was indicated. Ideally, conservative clinical therapy should be offered in a primary healthcare setting, without a need for further evaluation.

Thus, referrals were regarded as inappropriate when the participant may have benefitted from receiving assistance at a primary center, as opposed to a referral. In our study population, 43% of referrals were regarded as inappropriate. Previous studies have revealed that depending on the medical specialty, 10%-45% of referrals by primary care health care professionals are inappropriate, from the point of view of specialists.^(20)^

In 2015, a commission from the *Lancet Journal* determined the urgent need to establish access to surgical care for populations in low- and medium-income countries.^(21,22)^ Globally, approximately 5 billion people are estimated to not have access to proper surgical care.^(23)^ Therefore, guaranteeing proper specialized referrals is paramount to adequately distributing resources and ensuring the continuity of surgical care for patients who truly require it.^(24)^

Regarding clinical specialties, both national^(6,25)^and international^(21,26)^ papers have evaluated referral appropriateness, from primary health care centers. As per those studies conducted in Brazil, the rate of referral inappropriateness is >40%. These findings, which mimic our own, may indicate that specialized referral protocols in the Brazilian SUS require prompt revision to lessen the burden on secondary and tertiary services and reduce delays in patient care.^(27,28)^

Reasons for inappropriate referral protocols include a lack of proper healthcare infrastructure or clinical guidance regarding referrals of potential surgical cases.^(24,29)^ This disparity may be particularly more pronounced in specialized examinations seldom performed by GPs, such as in the case of vascular surgery.

Based on the findings of our study, the management of PAD deserves revision; because 69% of the participants with PAD were unnecessarily referred to the quaternary vascular surgical service. Of the evaluated vascular diseases, higher rates of inappropriate referrals were observed. General practitioner inexperienced in palpating peripheral pulses and the absence of protocols establishing signs of critical limb ischemia may partially elucidate these findings.

However, this phenomenon is not exclusive to Brazil. A study conducted in the United Kingdom evaluating referral letters to a vascular surgical service has revealed that in 90% of cases, the absence of peripheral pulses or signs of critical limb ischemia has not been mentioned.^(30)^

Moreover, participants were referred to the incorrect specialty. In our study, most inappropriate referrals occurred in cases of an ascending aortic aneurysm, a condition that should be treated by a cardiac surgeon.

Our findings revealed that the rate of surgical treatment for patients referred from a primary healthcare setting had been steadily decreasing over the years. Participants with PAD and carotid arterial diseases had lower surgical treatment rates (18.4 and 18%, respectively). We believe that misinformation regarding surgical indications and a delay in patient access to specialized care, resulting in disease progression, may explain these findings.

The unavailability of complementary examinations at the first appointment, in addition to inadequate or old examinations contributed to delays in treating patients.

For instance, regarding less complex procedures, patients with varicose veins who have already undergone a venous duplex scan can be referred for surgery at the first outpatient visit. Patients who arrive without a scan may require months for the completion thereof. Additionally, another appointment is required, to evaluate the results, and to perform a preoperative assessment.

The overburden of specialized services for patients with low-complexity conditions limits the proper operation of the system, by delaying or hindering access to patients with a real need for specialized care. This frequently results in disease progression, thus burdening emergency care units and increasing disease mortality and morbidity.

In Brazil, population studies have demonstrated that the following procedures are performed in an urgent setting: 1) approximately 62% of procedures for endovascular treatment of descending aorta aneurysms,^(31)^ 2) 68% of conventional treatments for thoracoabdominal aortic aneurysms,^(32)^ and 3) 52% of all procedures for the treatment of infrarenal aortic aneurysms.^(33)^

Should the referral system have functioned more promptly and efficaciously, a considerable number of these patients could have been treated in an elective setting, thus reducing surgical morbidity and mortality.

Several strategies have been proposed to address these concerns. During the coronavirus (COVID)-19 pandemic, digital health options, such as telemedicine, have gained popularity as options for care. Several initiatives, including those in Brazil, have been developed to incorporate these tools to facilitate healthcare access.^(34,35)^ As of 2022, the Federal Council of Medicine has regulated telemedicine in Brazil.^(36)^ This has introduced the potential of large-scale remote care and reduced number of inappropriate referrals.

Particularly prior to the COVID-19 pandemic, digital health methods have been proposed and studied as alternatives to patient care in vascular surgery, with promising results in optimizing referrals to specialized centers. Patients with nonvascular complaints or those who could be conservatively followed up are guided and followed up, using digital consultations, dispensing with an in-person appointment.^(37,38)^ Subsequent to the COVID-19 pandemic, these digital options have become particularly more widespread, permitting the surveillance of more complex conditions, such as aortic disease.^(39,40)^

Digital health and telemedicine may play important roles in reducing the patient burden on tertiary services. The vascular surgical service permits the periodic follow-up of patients with claudication; surveillance of aneurysmal and carotid arterial disease; and guidance of clinical management, until interventional therapy is indicated. This arrangement may reduce the need for specialized services, lower maintenance costs, and expedite the care of patients requiring specialized surgical care.

Specialist assessment remains the best option for determining the best treatment for a patient, particularly in areas where GPs are less proficient, as with vascular surgery. Therefore, the definition of an inappropriate referral is complex. We chose to characterize “inappropriate” referrals as cases with no indication or inclination for surgery.

Thus, clear referral protocols and the use of new tools, such as telemedicine, which allows contact with the specialist at a distance, lessens the demand without the need for a face-to-face assessment. This would result in reducing queues and optimizing the use of human and financial resources.

## CONCLUSION

The rate of referral appropriateness to specialized vascular care from primary care settings is low. This may represent a subutilization of tertiary surgical services, with low rates of surgical treatment. Digital healthcare options may present an alternative in reducing the patient burden on tertiary levels of care.
